# Automatic Peak Selection by a Benjamini-Hochberg-Based Algorithm

**DOI:** 10.1371/journal.pone.0053112

**Published:** 2013-01-07

**Authors:** Ahmed Abbas, Xin-Bing Kong, Zhi Liu, Bing-Yi Jing, Xin Gao

**Affiliations:** 1 Computer, Electrical and Mathematical Sciences and Engineering Division, King Abdullah University of Science and Technology, Thuwal, Saudi Arabia; 2 Department of Statistics, Fudan University, Shanghai, China; 3 Department of Mathematics, Faculty of Science and Technology, University of Macau, Taipa, Macau; 4 Department of Mathematics, Hong Kong University of Science and Technology, Kowloon, Hong Kong; University of Rome, Italy

## Abstract

A common issue in bioinformatics is that computational methods often generate a large number of predictions sorted according to certain confidence scores. A key problem is then determining how many predictions must be selected to include most of the true predictions while maintaining reasonably high precision. In nuclear magnetic resonance (NMR)-based protein structure determination, for instance, computational peak picking methods are becoming more and more common, although expert-knowledge remains the method of choice to determine how many peaks among thousands of candidate peaks should be taken into consideration to capture the true peaks. Here, we propose a Benjamini-Hochberg (B-H)-based approach that automatically selects the number of peaks. We formulate the peak selection problem as a multiple testing problem. Given a candidate peak list sorted by either volumes or intensities, we first convert the peaks into 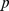-values and then apply the B-H-based algorithm to automatically select the number of peaks. The proposed approach is tested on the state-of-the-art peak picking methods, including WaVPeak [1] and PICKY [2]. Compared with the traditional fixed number-based approach, our approach returns significantly more true peaks. For instance, by combining WaVPeak or PICKY with the proposed method, the missing peak rates are on average reduced by 20% and 26%, respectively, in a benchmark set of 32 spectra extracted from eight proteins. The consensus of the B-H-selected peaks from both WaVPeak and PICKY achieves 88% recall and 83% precision, which significantly outperforms each individual method and the consensus method without using the B-H algorithm. The proposed method can be used as a standard procedure for any peak picking method and straightforwardly applied to some other prediction selection problems in bioinformatics. The source code, documentation and example data of the proposed method is available at http://sfb.kaust.edu.sa/pages/software.aspx.

## Introduction

Many computational bioinformatics methods generate a large number of predictions for the correct solution to a problem among which are both true and false predictions. Such predictions are usually sorted according to certain confidence scores. For instance, *ab initio* protein structure prediction methods sample tens of thousands of three-dimensional models. The energy values are calculated for each model based on a given energy function, where lower values likely indicate better models. Another example is the protein function annotation problem in which the amino acid sequence or the domain architecture of a protein is given and the Gene Ontology (GO) terms selected from among some 30,000 are used to annotate the function.

In nuclear magnetic resonance (NMR)-based protein structure determination, thousands of peaks are routinely predicted from the input spectra in which there are usually tens to hundreds of true signals. The peaks are sorted according to either their intensities or estimated volumes. Both means of sorting, based on computational methods, have common properties. First, a large number of predictions are generated. Second, the predictions are scored by the scoring functions of the methods. However, the scoring functions are not powerful enough to distinguish true predictions from the false ones. Third, it is important to discover most of the true predictions while maintaining a reasonably low false positive rate. Therefore, it is crucial to know how many predictions should be selected in such scenarios.

Peak picking is one of the key problems in NMR protein structure determination process [3]–[5]. The problem is defined as follows: given any NMR spectrum or a set of spectra, select the true signals, i.e., peaks, while filtering the false ones. Typically, true peaks are assumed to have Gaussian-like shapes and high intensities so that they can be easily differentiated from false ones. However, there are two main factors that make the peak picking problem difficult. On the one hand, depending on the quality of the protein sample, the property of the target protein and local dynamics, there can be a number of weak peaks, i.e., peaks with low intensities or volumes. That is, if we sort the predicted peaks by volumes or intensities, there is no clear cutoff threshold to distinguish true peaks from false ones. These peaks are difficult to identify even by manual processes. This is why computational methods are useful. On the other hand, due to the various sources of noise in NMR spectra, such as water bands and artifacts, false peaks can have high intensities or volumes. The group of sorted peaks is therefore comprised of a mixture of true peaks and false ones, where most of the true peaks tend to be ranked higher with a few strong, false peaks also included. It is extremely difficult, if not impossible, to select only the true peaks and eliminate all the false ones. In NMR structure determination, a missing true peak may cause all the follow-up procedures to fail, whereas a false peak can still be eliminated later [6]–[9]. Therefore, an ideal method should identify almost all the true peaks while maintaining reasonably high precision.

The peak picking problem has been studied for more than two decades. A variety of computational methods have been proposed [1], [2], [10]–[19]. The existing methods can be classified into two categories according to the de-noising method. Included in the first category are hard threshold-based approaches. For instance, PICKY [2] assumes that the noise is white Gaussian and estimates the noise level in small regions that do not contain signals. The data points that have lower intensities than the estimated noise level are eliminated from the spectra. Singular value decomposition is applied to the connected components of the remainder of the spectra to yield one-dimensional lineshapes. The peaks are identified in each lineshape and sorted according to the intensity values. The higher the intensity is, the greater the confidence that it is a true peak. However, the hard threshold-based methods cannot detect weak peaks that are embedded in the noise. In the second category are soft threshold-based approaches, which do not eliminate any data point from the spectra. We recently proposed WaVPeak [1] to overcome the bottleneck in the hard threshold-based methods. WaVPeak applies the high-dimensional version of the Daubechies 3 wavelet [20] to smooth the given spectra. The shapes of true peaks become sharper and smoother. A brute-force method is used to identify all the local maxima in the smoothed spectra. In contrast to PICKY, the peaks are sorted according to their estimated volumes by WaVPeak. We have found that volume significantly outperforms intensity in distinguishing true peaks from false ones.

However, the existing peak picking methods are not able to determine automatically how many peaks among many to identify in order to include most of the true peaks. This number should be large enough to include as many true peaks as possible, and in the meanwhile small enough to achieve relatively high precision. In PICKY, the default number of peaks to return is 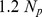, where 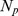 is the length of the protein. In [1], WaVPeak is mainly compared with PICKY on the top 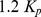 peaks, where 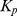 is the number of manually identified peaks, which is unknown for a new target protein. However, such fixed number-based approaches do not take the distribution of peaks into consideration. For instance, if there is a spectrum that is very noisy or has a large number of artifacts, there can be many strong but false peaks, which are identified along with the true ones. Many true peaks will not be selected if 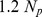 or 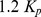 is used. No matter how powerful the peak picking method is, it is crucial to cleverly determine the number of peaks to be selected. Otherwise, true peaks will be eliminated even if they have been identified by the methods.

In this paper, we propose a Benjamini-Hochberg (B-H)-based approach for the peak picking problem. We first cast the peak selection problem into a multiple testing problem [21]. Because there is no clear cutoff threshold for intensities or volumes, we calculate the 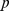-value for each peak. The number of peaks to be selected is then automatically determined by the B-H-based algorithm. We demonstrate that the proposed method significantly outperforms the fixed number-based method on selecting the true peaks from the predictions by the state-of-the-art peak picking methods, including WaVPeak and PICKY.

## Methods

Our goal is to develop a method to help us to determine how many peaks to select among candidate peaks that number usually in the order of several hundreds. Each candidate peak can be considered as a null hypothesis, where each false peak is a true null hypothesis and each true peak is a false null hypothesis. Therefore, the goal is to simultaneously test all the hypotheses and to reject as many false null hypotheses as possible. This is a multiple testing problem, which has received much attention in the literature (see, e.g., [22]). One prominent solution to multiple testing problem was proposed by Benjamini and Hochberg [23]. We first describe how to cast our problem into that framework.

### A Quick Review of Benjamini-Hochberg Method

We wish to test 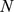 null hypotheses:
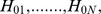
on the basis of a data set 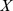. We have some decision rule 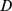 that rejects or accepts each of the above 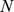 cases (e.g., decides if the 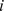
th candidate peak is a true peak or a false peak). The data set 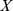 consists of



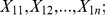


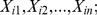


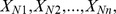
where 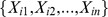 are a random sample from the 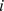
th population (e.g. intensities or volumes in a neighborhood of the 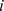
th candidate peak). We assume that our decision rule, 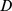, produces a 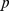-value, 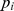, for each case, 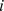 (we will discuss several different ways of calculating such 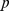-values later). Therefore, 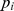 has a uniform distribution if 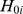 is correct,



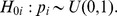



Intuitively, if the 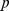-value, 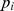, is small enough, 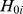 will be rejected. In fact, the usual Bonferroni procedure [24], [25] rejects 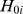 whenever
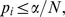
where 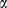 is the significance level. This is typically a very conservative procedure, particularly when 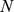 is large, because it does not reject as many null hypotheses as it should. In other words, it tends to have a low discovery rate.

**Figure 1:**
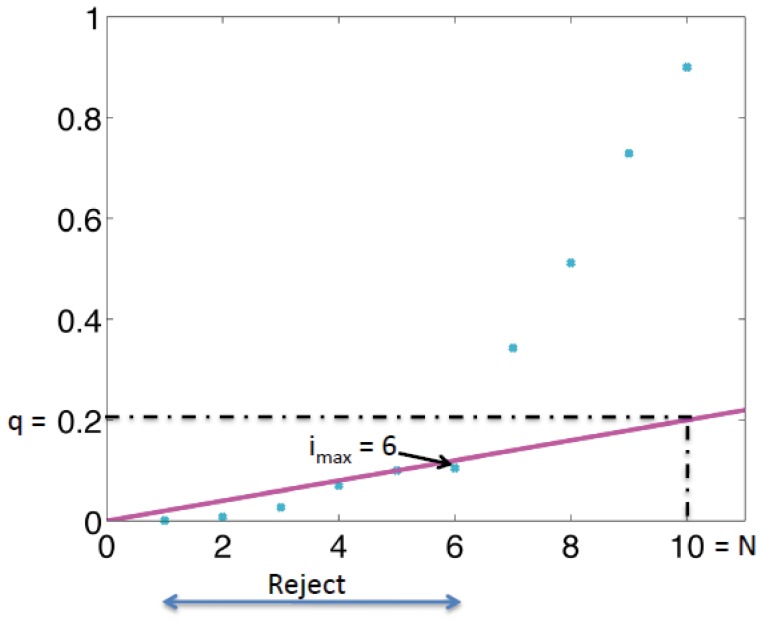
Illustration of the Benjamini-Hochberg procedure. In this example, the number of hypotheses (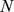) is 10 and the false discovery proportion (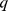) is 0.2. The largest index of the hypotheses that is below the line is 6 (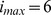). Therefore, the first six hypotheses are rejected as the predicted peaks.

To improve the discovery rate, Benjamini and Hochberg (1995) proposed an algorithm based on ordered 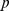-values:
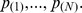



The Benjamini and Hochberg (B-H) algorithm uses the following rule: for a fixed value of 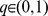, referred to as the control rate, let 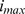 be the largest index for which
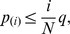
and reject 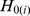, the null hypothesis corresponding to 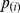, if



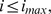
accepting 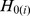 otherwise. Figure 1 illustrates how the B-H procedure works.

Benjamini and Hochberg proved the following result [23], which justified their procedure.

#### Theorem

For independent test statistics, the B-H algorithm controls the expected false discovery proportion (FDP) at 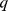:
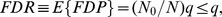
where 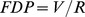, 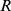 is the number of cases rejected, 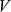 is the number of those that are actually null, and 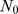 is the number of true null hypotheses.

Clearly, the above FDP control attempts to keep the number of false discoveries under control, and in a sense to keep the precision above a certain level. A good procedure should have as high recall rates as possible with prescribed high precision (or low FDP).

### Applying the B-H Procedure to the Peak Picking Problem

We will cast the NMR peak picking problem into the multiple testing framework. In WaVPeak (or PICKY), after data cleaning at the first stage by wavelet smoothing (or by hard thresholding), 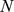 potential peaks are identified. We wish to test that, for each 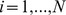,
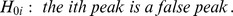
against



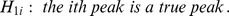



We can view each candidate peak and its surroundings as one population. We have a random sample of intensities, 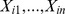 from the 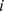
th population. The sample size 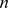 depends on which method is adopted. For WaVPeak, we have 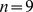 if we use a rectangular neighborhood of length 1 in 2D spectra, such as ^15^N-HSQC; for PICKY, we have 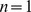 since we only use one intensity at each candidate peak.

We implement the B-H procedure below in two steps.

- Step I: calculating 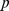-values.

For WaVPeak and PICKY, we use volume (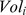) and intensity (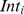) around the 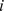
th candidate peak as the test statistics, respectively. Our decision rule is to reject 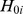 if 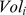 or 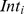 is large, respectively. The corresponding 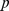-values are
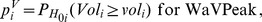


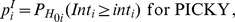
where 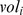 and 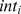 are observed values of 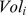 and 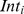.

- Step II: applying the B-H procedure at 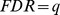.

Rank the 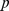-values 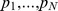 obtained from Step I in ascending order, and denote the ordered 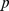-values as 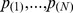. We can then plot 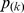 vs 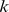, and apply the B-H procedure.

### Calculation of *P*-values

We now explain how to calculate p-values 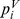 and 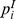 in Step I above. We assume that the observations from different peaks are independent, and that true peaks and false peaks are from two different normal distributions. Then we can rewrite the above testing problem as
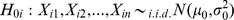
against



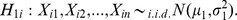



Typically, the mean intensity 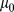 from false peaks is much smaller than the mean intensity 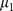 from true peaks, usually written as 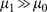. However, 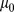 may not be zero, and can be estimated from weak intensities. For variances, we typically have 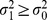.

The reason why 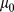 is small (compared with 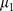) but not zero is due to how the candidate peaks are selected. In WaVPeak and PICKY, the volumes and intensities are calculated for a grid of points, respectively, those below certain thresholds are discarded, and the remaining ones are retained as candidate peaks. Therefore, the volumes and intensities for those candidate peaks should all have mean volumes and intensities above the thresholds.

To calculate 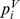 and 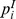, we need to standardize by subtracting the mean, 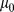, and divide the standard deviation (s.d.), 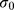, under 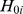
’s. Due to the different data structures of WaVPeak (volumes) and PICKY (intensities), they are considered separately below.

### Calculation of 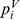


In WaVPeak, the test statistic 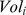 is the approximate volume under the 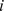
th candidate peak: 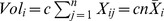, where 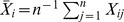 and 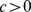 is some constant. Then, the 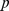-value is
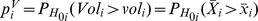


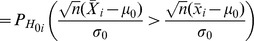


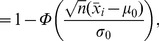
(1)where 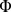 is the standard normal distribution. The mean/median, 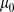, and variance, 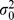, of the false peaks are unknown, which can be estimated by the sample median and sample variance of the false peaks, respectively. To do this, we need to have a rough idea of where those false peaks are located. It has been observed that the number of true peaks of a protein, 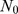, is always less than 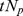, where 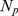 is the length of that protein and 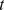 is the expected number of peaks per residue for the corresponding spectrum. For instance, for 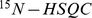, 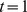; for 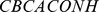, 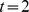. Almost all true peaks are ranked in the top 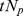 candidate peaks by volume in WaVPeak, while the remaining 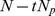 candidate peaks are mostly false peaks, from which we can estimate 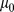 and 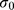.

To be more specific, for 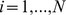, let 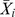 and 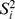 denote the sample mean and variance for the 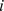
th candidate peak; and 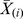 and 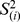 the ordered sample means and variances, respectively. Then 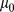 and 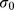 can be estimated by the medians of the 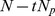 smallest 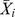 and 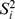:
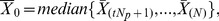
(2)

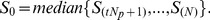
(3)


### Calculation of 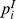


In PICKY, the test statistic is the intensity, 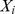, at the 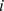
th single peak. Here, 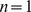. Its 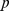-value can be calculated similarly to that in WaVPeak, giving
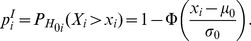
(4)


Although we could use the same estimators of 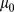 and 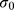 as above, we will propose some different ones for PICKY due to its unique features. It has been demonstrated that the intensity of a single peak point is a much less reliable confidence score than the volume of the peak [1]. It is thus expected that the intensity curves are mixed up by fewer true peaks and more false peaks. Therefore, the median of 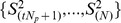 may no longer be accurate because the median may very likely come from a true peak. On the other hand, replacing the median by the minimum in (2) and (3) should produce better estimators of 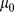 and 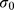, respectively, which turns out to be true for less reliable confidence scores (data not shown). Based on these considerations, we propose to estimate 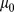 and 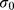 in PICKY respectively by
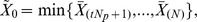


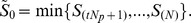



## Results

We evaluated the performance of the proposed methods on the peaks predicted by WaVPeak and PICKY. The same dataset as the one used by both [1] and [2] was used as the benchmark dataset, the most comprehensive dataset available for the peak picking problem. The dataset covers a wide range of spectrum types, including 2D ^15^N-HSQC, and 3D HNCO, HNCA, HNCACB and CBCA(CO)NH, which were extracted from the spectrum sets of eight proteins (TM1112, YST0336, RP3384, ATC1776, CASKIN, HACS1, VRAR, and COILIN).

We first demonstrate how our method performed when a more reliable confidence score is available, i.e., the estimated volumes of the peaks predicted by WaVPeak. We then present the performance of the method when a less reliable confidence score is available, i.e., the single intensity values of the peaks provided by PICKY. We finally demonstrate how to combine the results of our method with both WaVPeak and PICKY, to further eliminate false positive peaks.

### Selecting WaVPeak Peaks

The B-H algorithm is first compared with a fixed number-based method, i.e., 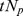, on selecting peaks predicted by WaVPeak. 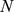 is set to 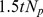. That is, the top 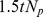 peaks predicted by WaVPeak are considered. The results are presented in Table 1, about which we make the following observations.

**Table 1:** Comparison of the missing peak rate of the fixed number-based method (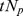) and the Benjamini-Hochberg (B-H) algorithm with 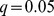 on the 32 spectra of the eight proteins in the benchmark dataset as picked by WaVPeak.

Spectra		^15^N-HSQC			HNCO			HNCA			HNCACB			CBCA(CO)NH		
Protein	Len	*tN_p_*	*B-H*	(*d*)	*tN_p_*	*B-H*	(*d*)	*tN_p_*	*B-H*	(*d*)	*tN_p_*	*B-H*	(*d*)	*tN_p_*	*B-H*	(*d*)
RP3384	64	7	7	(0)	0	0	(0)	12	12	(0)	–	–		8	8	(0)
CASKIN	67	6	2	(67)	22	15	(32)	–	–		38	41	(−8)	10	10	(0)
VRAR	72	3	3	(0)	7	7	(0)	–	–		31	32	(−3)	18	18	(0)
HACS1	74	7	2	(71)	8	6	(25)	–	–		14	15	(−7)	8	6	(25)
TM1112	89	8	2	(75)	–	–		6	6	(0)	8	7	(13)	9	2	(78)
COILIN	98	3	0	(100)	16	17	(−6)	–	–		24	25	(−4)	28	20	(29)
ATC1776	101	7	5	(29)	8	7	(13)	19	17	(11)	–	–		25	24	(4)
YST0336	146	2	2	(0)	6	3	(50)	11	10	(9)	–	–		17	13	(24)
*Average*		5.4	2.9	(43)	9.6	7.9	(16)	12	11.2	(5)	23	24	(−2)	15.4	12.6	(20)
*SD_ave_*		2.2	2.0		6.7	5.7		4.6	3.9		10.9	12.0		7.4	7.0	
*Pre_ave_*		84	77		77	73		83	76		64	71		72	67	

Column 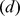 is the relative improvement of the missing peak rate of B-H over 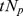. All values except the last two rows are the missing peak rates. The “
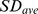
” row lists the standard deviations of the missing peak rates for the corresponding columns, demonstrating the robustness of different methods. The last row is the average precision value. All values are given in percentage.

**Figure 2:**
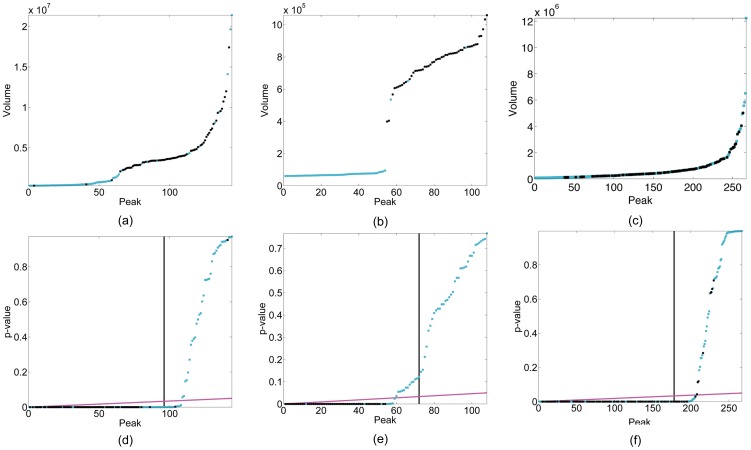
Original volume curves and the corresponding p-value curves. (a) and (d): sorted volume curve (a) and the corresponding p-value curve (d) of peaks predicted by WaVPeak on the 2D ^15^N-HSQC spectrum of the protein ATC1776; (b) and (e): sorted volume curve (b) and the corresponding p-value curve (e) of peaks predicted by WaVPeak on the 3D HNCO spectrum of the protein VRAR; (c) and (f): sorted volume curve (c) and the corresponding p-value curve (f) of peaks predicted by WaVPeak on the 3D CBCA(CO)NH spectrum of the protein COILIN. In all figures, true peaks are shown in black and false ones are shown in cyan. In (d), (e) and (f), the decision boundaries of 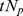 and B-H procedure are shown in black and magenta, respectively.

- The B-H algorithm significantly outperforms the 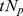-based method in terms of the average missing peak rates, i.e., the percentage of true peaks that are not selected. On six out of the 32 spectra, the B-H algorithm reduces the 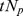-based method on the missing peak rate by more than 50%. One exception is HNCACB, where the B-H algorithm is slightly worse than the 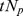-based selection in the missing peak rate (but better in precision); however, this can be easily rectified by increasing the FDR 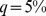 to 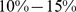, which is commonly adopted in practice. Overall, the B-H algorithm is much more sensitive and stable than the fixed number-based method. It is noticeable that the improvement in the sensitivity is at the cost of the reduced precision. This is expected because the B-H algorithm does not change the order of the sorted candidate peaks. Instead, it provides a good tradeoff that prefers higher sensitivity by selecting a cutting point in the list of the sorted peaks.
- As expected, the fixed number-based method is not stable. It performs well on some spectra (e.g. RP3384), but poorly on the others (e.g., TM1112). This is further verified by its larger standard deviations. The reason is that such a method does not take the properties of the input spectra into consideration. For instance, for a very noisy spectrum with weak signals, there can be many false peaks sorted amongst the true ones (e.g., Figures 2(a) and 2(c)). Thus, by taking a fixed number of peaks, there is no way one can ensure that the true peaks are included.
- Reduction on the missing peak rate of B-H over 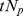 can reach as high as 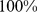, indicated in column 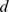. These improvements mostly occur in the weak peaks, which are the most difficult to find. Since there are not many weak peaks to start with, improvements measured by *relative* missing peak rates (i.e., weak signals found/all weak signals) are very high, even though those measured by absolute missing peak rates may not always appear.

It is noticeable that all the missing peak rates in Table 1 are the results by comparing to the “expected” peak lists of the spectra. The “expected” peak lists were generated by NMR labs by combining information from large sets of spectra. It is thus likely that an expected peak does not exist in some spectra, especially the noisy ones, such as HNCACB and CBCA(CO)NH. In practice, higher recall rates (lower missing peak rates) than those reported here can be expected.

Figure 2 shows several representative examples of how different selection methods work. We make several remarks.

- It can be difficult to set a cutoff point from the original volume curves in Figures 2(a)–2(c) to separate true peaks from false ones. The best thing the fixed number-based methods can do is to take a random guess. For example, the 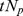-based selection method overestimates the number of peaks to be selected for a less noisy spectrum as shown in Figure 2(e), but significantly underestimates the number of peaks to be selected for a noisier spectrum as shown in Figure 2(f).
- The B-H algorithm works consistently well on the 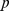-value curves. As shown in Figure 2, after converting the volumes to 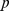-values, strong true peaks with high volumes are dragged down to the 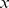-axis, i.e., the 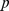-values are almost equal to zero. Most of the weak true peaks with low volumes are also dragged to the 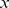-axis, making it possible to identify them in the 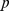-value curves. For instance, two of the three weak peaks with low volumes in Figure 2(a) are dragged down to the 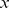-axis, and thus selected by the B-H algorithm. Note that the 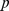-value does not change the volume order of the peaks. Instead, it provides a much better curve so that the weak peaks can be possibly selected.

### Selecting PICKY Peaks

We then evaluated the performance of the proposed method with a less reliable confidence score, i.e., the intensity value of PICKY. PICKY has a default noise level threshold [2], which sometimes causes insufficient numbers of predicted peaks. For fair comparison purposes, we lowered the noise level threshold of PICKY until it generated more than 1.5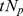 peaks.

**Figure 3:**
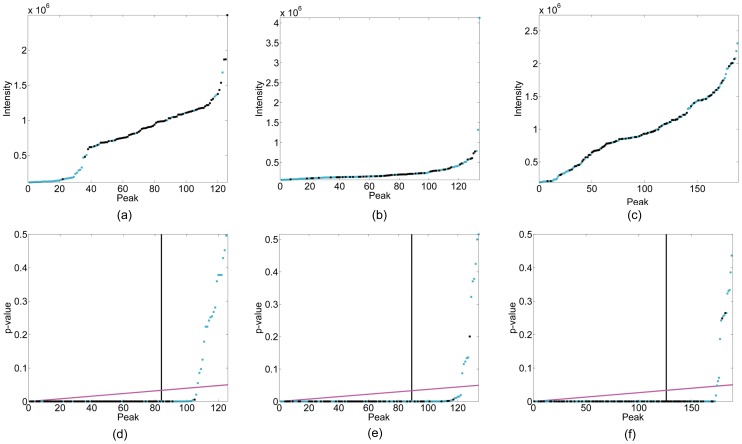
Original intensity curves and the corresponding p-value curves. (a) and (d): sorted intensity curve (a) and the corresponding p-value curve (d) of peaks predicted by PICKY on the 2D ^15^N-HSQC spectrum of the protein TM1112; (b) and (e): sorted intensity curve (b) and the corresponding p-value curve (e) of peaks predicted by PICKY on the 3D HNCO spectrum of the protein COILIN; (c) and (f): sorted intensity curve (c) and the corresponding p-value curve (f) of peaks predicted by PICKY on the 3D CBCA(CO)NH spectrum of the protein RP3384. In these figures, true peaks are shown in black and false ones are shown in cyan. In (d), (e) and (f), the decision boundaries of 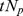 and the B-H procedure are shown in black and magenta, respectively.

Table 2 presents the performance of the proposed method on selecting peaks predicted by PICKY. Similar conclusions to those about WaVPeak can be made here. For instance, the B-H method consistently and significantly outperforms the fixed number-based method. There are seven spectra on which the B-H algorithm reduces the missing peak rate of the 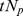-based method by at least 50%. Six of these spectra have original recall rates that were already higher than 90%. There are two spectra, HNCO of COILIN and CBCA(CO)NH of RP3384, on which the absolute improvements are greater than 15% with highest being 26%. As shown in Figures 3(b) and 3(c), the original intensity curves for these two spectra are continuous and smooth. It is difficult to identify a cutoff point between true peaks and false ones on such curves. Many false peaks are sorted amongst the true ones. After converting the intensity values into p-values, most of the true peaks are dragged down to the 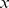-axis, i.e., they have very small p-values. The 5% slope is then able to select most of the true peaks. In the two cases, fewer than three true peaks are not selected and true peaks are almost the last ones selected by the B-H algorithm.

**Table 2:** Comparison of the missing peak rate of the fixed number-based method (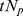) and the Benjamini-Hochberg (B–H) algorithm with 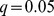 on the 32 spectra of the eight proteins in the benchmark set picked by PICKY.

Spectra	^15^N-HSQC			HNCO			HNCA			HNCACB			CBCA(CO)NH		
Protein	*tN_p_*	*B-H*	(*d*)	*tN_p_*	*B-H*	(*d*)	*tN_p_*	*B-H*	(*d*)	*tN_p_*	*B-H*	(*d*)	*tN_p_*	*B-H*	(*d*)
RP3384	6	4	(33)	0	0	(0)	13	13	(0)	–	–		36	10	(72)
CASKIN	2	2	(0)	22	15	(32)	–	–		33	38	(−15)	10	9	(10)
VRAR	7	7	(0)	9	9	(0)	–	–		31	31	(0)	19	19	(0)
HACS1	5	2	(60)	6	4	(33)	–	–		18	17	(6)	9	6	(33)
TM1112	8	1	(88)	–	–		6	6	(0)	8	7	(13)	9	2	(78)
COILIN	4	0	(100)	19	3	(84)	–	–		24	24	(0)	33	20	(39)
ATC1776	5	4	(20)	8	5	(38)	19	18	(5)	–	–		28	26	(7)
YST0336	2	2	(0)	6	3	(50)	12	10	(17)	–	–		17	12	(29)
*Average*	4.9	2.7	(38)	10.0	5.6	(34)	12.5	11.7	(6)	22.8	23.4	(1)	20.1	13.0	(34)
*SD_ave_*	2.0	2.0		7.2	4.6		4.6	4.4		9.1	10.8		10.3	7.5	
*Pre_ave_*	85	69		77	67		83	70		65	69		68	60	

Column 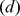 is the relative improvement of the missing peak rate of B-H over 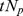. All values except the last two rows are the missing peak rates. The “
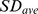
” row lists the standard deviations of the missing peak rates for the corresponding columns, which demonstrates the robustness of different methods. The last row gives the average precision values. All values are given in percentage.

### Eliminating False Peaks

The proposed B-H algorithm automatically determines how many peaks we should select from the candidate peak lists that are sorted according to the confidence scores of different methods. Therefore, the more true peaks it includes, the greater the possibility that it also includes false ones. This possibility is verified by the relatively low precision values in Table 1. The selected false peaks usually have larger volumes (or even much larger volumes) than the true ones. This can be caused by a variety of reasons, such as water bands, artifacts and side-chains. It is thus very difficult to eliminate them from a single spectrum. An effective way to eliminate false peaks is to use spectra that share same atoms to “cross-reference” the peaks [2].

The goal of such cross-referencing is to eliminate as many false peaks as possible, while maintaining as many true peaks as possible. Among the commonly used NMR spectra, ^15^N-HSQC is the most sensitive and reliable one. It is often used as the root spectrum by NMR spectroscopists. If ^15^N-HSQC is not available, HNCO is usually considered to be the root, especially in non-linear acquisition mode. If other types of spectra are used to cross-reference ^15^N-HSQC, the recall will be significantly decreased. Therefore, we used a consensus method to refine the peaks selected for ^15^N-HSQC. Both WaVPeak and PICKY were used to pick peaks for the ^15^N-HSQC spectra of the eight proteins. The two candidate peak lists were then selected by the proposed B-H algorithm. Only the peaks that appeared in both selected peak lists were kept as the consensus peak list for ^15^N-HSQC. As shown in Table 3, the consensus method retained all the true peaks while increasing the precision by 13% on average. The consensus peak list was then used to refine all the other peak lists of WaVPeak that were selected by the proposed B-H algorithm. The reason we used the peak lists of WaVPeak was that WaVPeak was shown to be more sensitive than PICKY on noisier spectra [1]. Table 3 shows that for all the spectra, most of the true peaks were maintained, and the precision values were significantly improved. F-score, which is the harmonic mean of precision and recall, suggests that the BH-based consensus method gives the best overall accuracy comparing to other methods, including PICKY, WaVPeak, B-H WaVPeak, and the consensus of PICKY and WaVPeak by simply considering the top 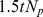 peaks from each method. On average, the BH-based consensus method was able to identify more than 88% of the expected true peaks, whereas less than 17% of the selected peaks were false ones.

**Table 3:** Comparison of the performance of different peak picking methods.

Spectra	^15^N-HSQC		HNCO		HNCA		HNCACB		CBCA(CO)NH		
Method	*Rec*	*Pre*	*Rec*	*Pre*	*Rec*	*Pre*	*Rec*	*Pre*	*Rec*	*Pre*	*F – score*
PICKY	93	81	89	74	88	74	60	78	72	66	77
WaVPeak	96	80	91	76	88	74	76	64	85	71	80
B-H (WaVPeak)	97	70	92	73	89	76	76	71	87	67	79
Consensus ( 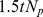 )	97	72	92	70	88	82	77	72	88	69	80
B-H (Consensus)	97	83	89	80	86	93	76	84	86	80	85

*Rec* stands for recall values and *Pre* stands for precision values. The recall and the precision values of PICKY and WaVPeak are taken from [1]. B-H (WaVPeak) is the WaVPeak peaks selected by the proposed B-H algorithm. Consensus (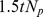) is the consensus of WaVPeak and PICKY by simply considering the top 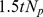 peaks from each method. B-H (Consensus) is the consensus of WaVPeak and PICKY by considering the top peaks that are determined by the proposed B-H algorithm. All the values are given as percentage.

Note that the performance of PICKY and WaVPeak in Table 3 was taken from that reported in [1], in which the top 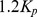 peaks were selected for comparison, where 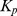, the number of true peaks that exist in the spectrum, was assumed to be known. The 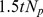 consensus method in Table 3 was done by considering the top 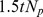 peaks of both PICKY and WaVPeak, which is much larger than the number of peaks used in [1]. This explains the significant drop of precision for the consensus method with respect to PICKY and WaVPeak.

**Figure 4:**
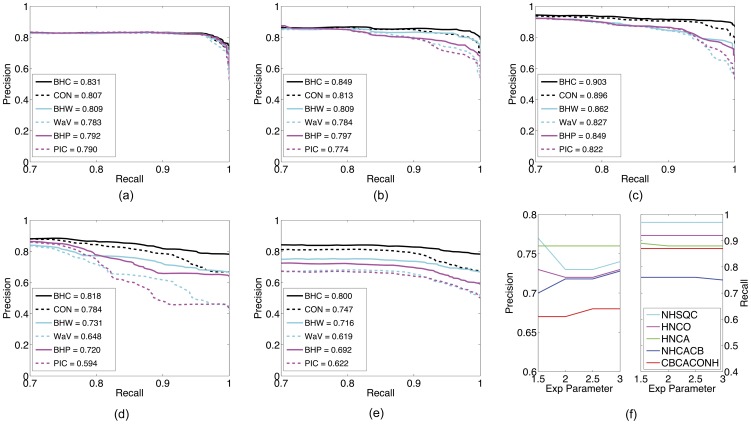
Precision-recall curves for different peak picking methods and sensitivity analysis of B-H WaVPeak. (a)–(e): precision-recall curves for different methods on ^15^N-HSQC, HNCO, HNCA, CBCA(CO)NH and NHCACB, respectively. The solid black curves are for B-H consensus method; the dashed black curves are for the 1.5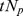 consensus method; the solid cyan curves are for B-H WaVPeak; the dashed cyan curves are for the original WaVPeak; the solid magenta curves are for B-H PICKY; and the dashed magenta curves are for the original PICKY. The relative area under curve (AUC) values are in legends, which are the area under curve over the total area of recall at least 0.7. (f): sensitivity analysis for different number of peaks. The precision and recall values of B-H WaVPeak are shown when 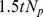, 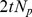, 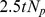 and 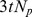 top peaks are used to calculate the p-values.

Figures 4(a)–(e) show the precision-recall curves of the six different peak picking methods on the five types of spectra. These six methods are PICKY, B-H PICKY, WaVPeak, B-H WaVPeak, 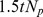 consensus and B-H consensus. For the sake of clearance, only the important parts of the curves, i.e., when recall is at least 0.7, are drawn. It is clear that B-H consensus always outperforms the five other methods. That is, at the same recall value, B-H consensus always has less proportion of false positive peaks. The 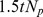 consensus is the second best method. This makes sense because the consensus methods, comparing to the other methods, combine information from different, relevant spectra. B-H WaVPeak and B-H PICKY consistently outperform WaVPeak and PICKY. Note that WaVPeak has been shown to be better than PICKY [1]. Thus, the improvement of B-H PICKY over WaVPeak is due to the use of our B-H algorithm. In practice, we suggest the users to use the B-H WaVPeak if high sensitivity is required or only one spectrum is available, and use the B-H consensus method if high tradeoff between precision and recall is needed or a set of relevant spectra is given.

We further studied the sensitivity of the B-H algorithm with respect to the parameter. In this paper, we have been using 1.5 as the parameter value in 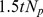. As shown in Figure 4(f), when the parameter value is changed to 2, 2.5 or 3, there is no significant change on both precision and recall.

## Discussion

A common issue in bioinformatics is that a large number of predictions are made by computational methods. These predictions contain both true predictions and false ones. In most problems, a fixed number of predictions is selected according to a certain confidence score. The confidence score, however, is not accurate enough to differentiate true predictions from false ones. Therefore, selecting a fixed number of predictions or thresholding by a fixed score usually sacrifices a lot of true predictions because it does not take the properties of the problem into consideration. We propose a general approach to partially resolve this issue. The original confidence score is first converted into 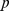-values, which have been demonstrated to have a much stronger distinguishing capability than the original confidence score. The Benjamini-Hochberg algorithm is then applied to select a self-adapted number of predictions according to the false discovery rate that we want to control. This approach provides a systematic way of selecting predictions of computational methods. We further demonstrate that the false predictions can be further eliminated by using consensus or cross-referencing approaches.

The proposed approach has a wide range of potential applications. For instance, in protein function annotation problems, the amino acid sequences or domain architectures of proteins are given, and the GO terms selected from among some 30,000 are used to annotate the function. Most of the existing methods estimate the probability for each GO term to annotate the given protein [26]–[29]. However, the number of GO terms that annotate a certain protein is unknown. Our approach can be directly applied to the protein function annotation problem such that the correct number of GO terms is selected.

Theoretically speaking, the sum of the false discovery rate and the precision should be one. However, the precision values of B-H WaVPeak and B-H PICKY are way below 0.95, as shown in Tables 1 and 2. This is due to the fact that the volume and the intensity used in the original WaVPeak and PICKY are not perfect measures to rank peaks. That is, although such measures contain information about peak properties, the information is far from complete or correct. As shown in Figures 2 and 3, many true peaks can have much lower volume or intensity than some false ones. In order to achieve the theoretical precision level, better measurements have to be used by the original peak picking methods. For instance, the symmetry of peak shapes can be considered as additional information to rank peaks [4].

We are currently incorporating the proposed method as a plug-in into the available NMR software, such as CCPN and NMRView [15]. The source code of the proposed method is available at http://sfb.kaust.edu.sa/pages/software.aspx.

### Conclusion

We have proposed a sensitive and robust approach to select peaks from automatic peak picking methods. The original peak confidence scores are first converted into 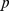-values. The Benjamini-Hochberg algorithm is then applied to select the number of peaks. In this paper, we demonstrated that the proposed approach worked consistently well using state-of-the-art peak picking methods. Therefore, this can be a potentially general approach to select a good number of candidates from a large set of predictions.
